# Non-invasive neuromodulation of the cervical vagus nerve in rare primary headaches

**DOI:** 10.3389/fpain.2023.1062892

**Published:** 2023-03-13

**Authors:** Maria Dolores Villar-Martinez, Peter J. Goadsby

**Affiliations:** ^1^ Headache Group, Wolfson CARD, Institute of Psychiatry, Psychology and Neuroscience, King's College London, London, United Kingdom; ^2^ NIHR King's Clinical Research Facility, SLaM Biomedical Research Centre, King's College Hospital, London, United Kingdom; ^3^ Department of Neurology, University of California, Los Angeles, Los Angeles, CA, United States

**Keywords:** VNS (vagus nerve stimulation), hemicrania, gammaCore, neurostimulation devices, rare headache

## Abstract

Primary headache disorders can be remarkably disabling and the therapeutic options available are usually limited to medication with a high rate of adverse events. Here, we discuss the mechanism of action of non-invasive vagal nerve stimulation, as well as the findings of the main studies involving patients with primary headaches other than migraine or cluster headache, such as hemicrania continua, paroxysmal hemicrania, cough headache, or short-lasting neuralgiform headache attacks (SUNCT/SUNA), in a narrative analysis. A bibliographical search of low-prevalence disorders such as rare primary headaches retrieves a moderate number of studies, usually underpowered. Headache intensity, severity, and duration showed a clinically significant reduction in the majority, especially those involving indomethacin-responsive headaches. The lack of response of some patients with a similar diagnosis could be due to a different stimulation pattern, technique, or total dose. The use of non-invasive vagal nerve stimulation for the treatment of primary headache disorders represents an excellent option for patients with these debilitating and otherwise refractory conditions, or that cannot tolerate several lines of preventive medication, and should always be considered before contemplating invasive, non-reversible stimulation techniques.

## Introduction

Migraine and cluster headache apart, the International Classification of Headache Disorders, 3rd edition (ICHD-3), contains other disabling primary headaches; among these are trigeminal-autonomic cephalalgias, including hemicrania continua and chronic paroxysmal hemicrania, or the short-lasting unilateral neuralgiform headache attacks, and more than a dozen primary headaches classified as “other,” such as cough headache, hypnic headache, or exertional headache (Table 1) (1).

**Table 1:** Main features of the primary headaches treated with nVNS, modified from the ICHD-3, International Classification of Headache Disorders, 3rd edition.

	Hemicrania continua	Paroxysmal hemicrania	SUNA	Primary cough headache
Frequency	Persistent	>5 attacks/24 h	>1 attack/24 h	Unspecified
Chrono-biology of headache	Present for >3 months	At least 20 attacks Episodic: At least two bouts lasting from 7 days to 1 year (when untreated) and separated by pain-free remission periods of >3 months Chronic: Occurring without a remission period or with remissions lasting <3 months, for at least 1 year	Unspecified number Episodic: At least two bouts lasting from 7 days to 1 year (when untreated) and separated by pain-free remission periods of >3 months Chronic: Occurring without a remission period or with remissions lasting <3 months, for at least 1 year	2 attacks
Duration	Constant	2–30 min	1–600 s	1 s–2 h
Description	Strictly unilateral, with exacerbations of moderate or greater intensity	Severe unilateral orbital supraorbital and/or temporal pain	Moderate or severe unilateral head pain, with orbital, supraorbital, temporal, and/or other trigeminal distribution and occurring as single stabs, series of stabs, or in a saw-tooth pattern	Sudden onset. Induced by and occurring only in association with coughing, straining, and/or Valsalva manoeuvre
Indomethacin response	Responds fully to therapeutic doses of indomethacin		NA	NA
Cranial-autonomic symptoms	At least one of the following, ipsilateral to the pain (a) conjunctival injection/lacrimation (b) nasal congestion and/or rhinorrhoea (c) eyelid oedema (d) forehead and facial sweating (e) miosis and/or ptosis If cranial-autonomic symptoms are absent, a sense of restlessness or agitation, or aggravation of the pain by movement is experienced.			NA
	Not better accounted for by another ICHD-3			

nVNS, non-invasive vagus nerve stimulation; NA, not applicable.

A majority of these headaches have a low prevalence rate, and the therapeutic armamentarium is usually limited to pharmacological options with a wide range of side effects, such as indomethacin or antiepileptic medications (2).

**Figure 1:**
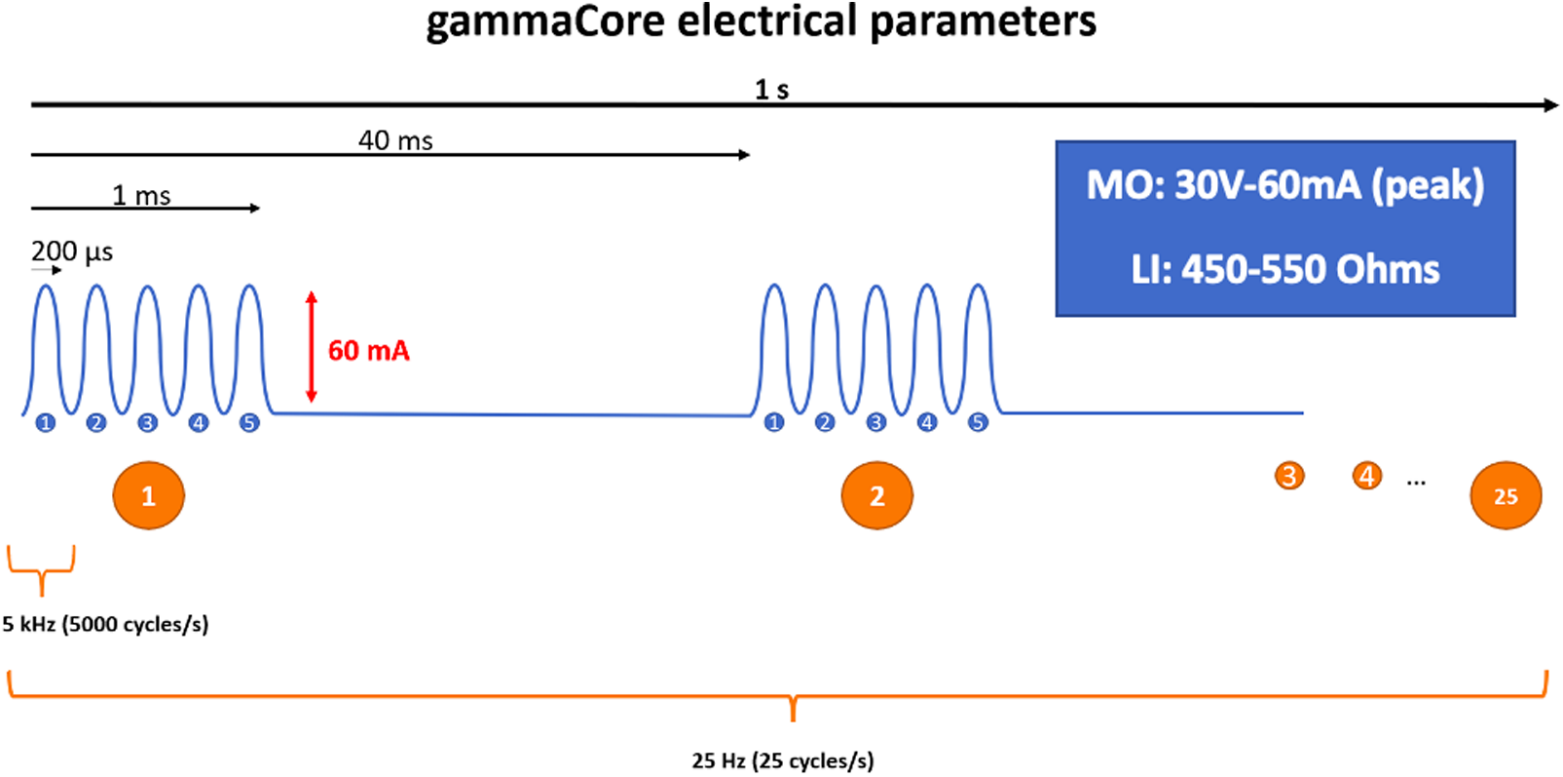
Schematic representation of the electrical parameters of the gammaCore device: 5 kHz sine wave burst lasting for 1 ms (five sine waves, each lasting 200 μs), repeated once every 40 ms (25 Hz), generating an output of 60 mA at 30 V. LI, load impedance; MO, maximum output.

Vagal nerve stimulation (VNS) was first investigated in 1990 for the treatment of intractable epilepsy (3, 4). In this review, we will cover findings related to non-invasive, transcutaneous cervical (nVNS). The United States Food and Drugs Administration approved gammaCore (Figure 1) for the acute and preventive treatment of migraine headache in adults and adolescents, adjunctive preventive in adults with cluster headache, and acute treatment in episodic cluster headache, and recently, also for adults with hemicrania continua and paroxysmal hemicrania (5).

## Literature review strategy

We performed a narrative review of the literature using PubMed, EMBASE, and Web of Science on September 2022. Keywords included primary headaches 3.2–4.10 from the ICHD-3 (1) AND “gammacore” OR “vagus nerve stimulation” and “pathophysiology” AND “gammacore” OR “vagus nerve stimulation.” Articles addressing the mechanism of action and translational research that help comprehending the pathophysiology of vagus nerve stimulation were included. The search included publications in English and Spanish. Individual case reports were included. Additional information was obtained from abstracts presented in international conferences. There were no exclusion criteria, given the low number of studies in this specific topic.

## Mechanism of action

The first time that electrical VNS was used in research was in an animal model in 1952, to demonstrate that the effects observed following carotid compression, spontaneous movements, were due to the neurogenic component and not to blood pressure change (6). Consistent with the neurovascular pathophysiology of primary headache disorders (7), the efficacy of VNS does not seem to involve vascular changes, as mentioned in a 2015 abstract of a pilot study (8).

**Figure 2:**
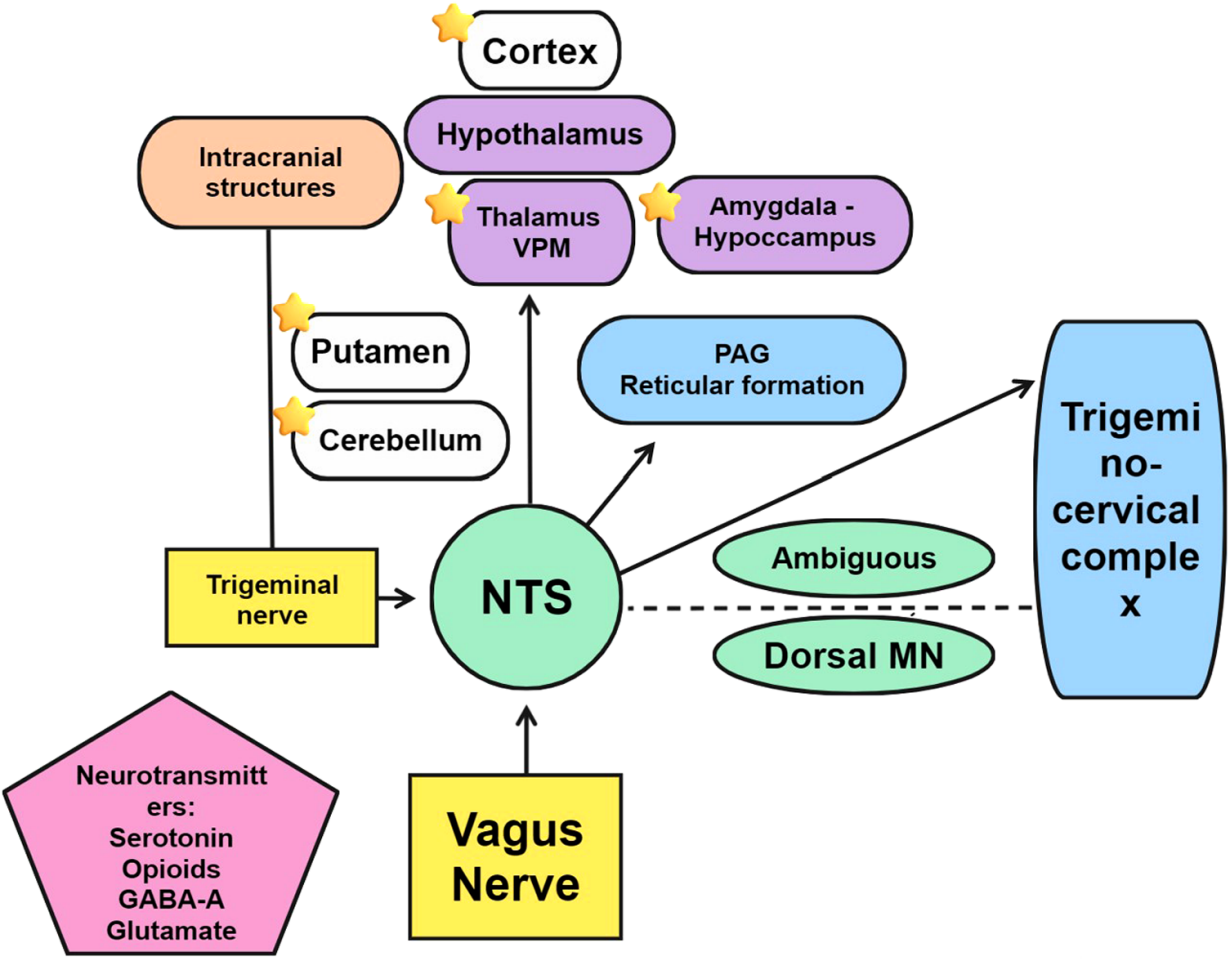
Structures and neurotransmitters involved in the mechanism of action of vagal nerve stimulation. Stars indicate changes in regional brain flow.

The mechanism of action of VNS in primary headache disorders has not been fully elucidated. Its effects are multiple and have been collated into four areas, namely, nociceptive modulation, neurotransmitter regulation, cortical spreading depression, and autonomic effects (9). The anatomical basis of the therapeutic effect of nVNS is likely to be the connection between the vagus nerve with relevant central nervous system structures (Figure 2). Among them, the key intersection in the medulla with the nucleus tractus solitarius (NTS) is important. The NTS is activated by nociceptive afferents from intracranial structures innervated by the trigeminal nerve (10) and projects in a topographic fashion to several higher structures, including medullary structures, such as the reticular formation or the periaqueductal grey. NTS also projects ipsilaterally to the parvicellular part of the ventral posteromedial thalamic nucleus (11) and connects with the caudal parts of the spinal trigeminal nucleus and thus the trigeminocervical complex (12).

The spinal part of the trigeminal nucleus and the other three vagal nuclei, the NTS, nucleus ambiguous, and dorsal motor nucleus, are highly interconnected (13, 14). The antinocifensive effect of VNS in rats can be inhibited by the administration of gamma aminobutyric acid A and serotonin receptor antagonists in the upper spinal cord, causing an effect similar to that of sumatriptan (15). The mechanism of action of VNS also involves other neurotransmitters, and among these is the opioidergic system (16), especially through the activation of delta opioid receptors (17).

Electrical stimulation of vagal afferents is capable of directly altering the response of trigeminal and trigeminothalamic neurons that have been stimulated with noxious inputs in the orofacial region of cats. These were wide-dynamic-range neurons that responded both to tactile stimuli and to painful heat stimuli. Continuous stimulation at 5 Hz, 3 ms, 2 mA inhibited a majority of the heat-induced neuronal responses, whereas one-third either remained unaffected or were facilitated. Intermittent stimulation before the test stimulus was assessed in A*δ* and C-fibres, delivering seven pulses at 333 Hz, 5 mA, in 200 ms, with similar results of inhibition or inactivity (18). In rats, the application of VNS for 24 h at 2 mA intensity, 20 Hz frequency, 0.5 ms pulse width, and a duty cycle of 20/18 s in ON/OFF, respectively, reduced the responses to formalin injected in trigeminal areas, as compared with sham stimulation (19). In another study, continuous VNS at 10 Hz suppressed trigeminal responses evoked by dural electrical stimulation in 48% of neurons; however, there was a 29.5% of neuron activity facilitated by VNS, and 22.5% was unresponsive to stimulation. This difference was first hypothesised to be due to the recruitment threshold of the different types of neurons, with rapidly conductive pathways needing a lower intensity of stimulation, and high intensity stimuli would activate slowly conducting pathways and promote an antinociceptive effect; however, it may be actually affected by descending pain control mechanisms (20). Indeed, the beneficial effect might not even be dependent on C-fibres (21, 22). Another study evaluating single pulses of VNS in dural and superior salivatory nucleus–evoked trigeminocervical firing found the beneficial effect to be dose-dependent and side-independent (23).

The central activity is further supported by studies assessing VNS in processes with higher structures implicated in pain processing. Activation of the trigeminal-autonomic reflex is significantly inhibited by nVNS, which involves several central structures, including the pontine nucleus, hypothalamus, or parahippocampal gyrus (24, 25).

Associated symptoms involving central sensitisation processes such as allodynia also respond to VNS stimulation, with a reduction in extracellular glutamate (26), and a modulation of the nociceptive withdrawal reflex is also possible following VNS, with an increase in the threshold to stimuli in animals and humans’ withdrawal effect on healthy subjects (27). Electrophysiological studies support a modulation of the pain matrix at cortical levels (28), and a direct or indirect cortical effect could also be inferred from animal studies showing a modulation of cortical spreading depression (29, 30).

Neuroimaging research supports the role of VNS in modulating the activity of brainstem and supratentorial structures. From studies in epileptic patients with cortical frontal and bitemporal in H_2_^15^O PET scan 2 milliamps 30 Hz 60 s, regional cerebral blood flow (rCBF) was increased in the ipsilateral structures of the left posterior cerebellum and left putamen and in the contralateral areas of the right middle temporal gyrus and right thalamus. Larger increases were seen in the cerebellum and thalamus in the patient with the best clinical response (ipsilateral cerebellum and contralateral thalamus) (31). Dorsal medulla and somatosensory cortex contralateral to the stimulation, hippocampus, and amygdala can also present alterations in rCBF in participants with different VNS stimulation patterns (32). More recent studies assessing transcutaneous VNS on healthy subjects using functional MRI found significantly activated primary vagal projections, remarkably, an activation of the NTS and deactivation of the spinal trigeminal nucleus, among others (33). At 15 V, cervical VNS is capable of eliciting a vagal response in more than 80% of participants (34).

An inflammatory component involving cytokines has been suggested to be part of the pathophysiology, outside the central nervous system, of other primary headaches (35, 36). Assuming a possible regulation of inflammatory mediators through a cholinergic pathway (37), one study found changes in cytokine levels in migraineurs following treatment with nVNS (38) that were, however, not significantly different in comparison with those of controls in previous studies (35, 36).

## Efficacy in headache

Several studies have demonstrated an excellent safety profile (39–42) and preliminary efficacy (39–41) in episodic and chronic migraine, with a reduction in headache intensity, severity, and duration of migraine attacks in three open label trials, two of them using the stimulation acutely. Participants with menstrual and menstrually related migraine (43) and migraine in adolescents (44) showed a reduction in the number of migraine days and analgesic consumption and successfully aborted attack, respectively, when treated with nVNS. Three randomised controlled studies included participants with cluster headache, who also presented a good tolerability to the device, with especially good efficacy in patients with the episodic form. The Prevention and Acute Treatment of Chronic Cluster Headache (PREVA), against standard of care, showed a significantly greater reduction in the number of attacks per week vs. controls (−5.9 vs. −2.1, respectively), ACT1 demonstrated response rates of 34.2% vs. sham 10.6% (*P* = 0.008) and ACT2 showed similar results, both in patients with the episodic form, with an efficacy of 48% vs. 6% in the sham group (*P *< 0.01) (45–49). nVNS has proved cost-effective in other trigeminal-autonomic cephalalgias, with a lower cost in combination with the standard of care acute treatment approach, higher benefits in terms of quality-adjusted life years, and potentially reduced visits to clinics and emergency departments (50).

### Efficacy of nVNS in indomethacin-sensitive headache disorders

Apart from cluster headache and migraine, nVNS has been reported to be effective in other primary headaches, especially indomethacin-sensitive headaches. However, given the rarity of these disorders, the quality of the study is usually power-limited, with a small number of participants given the challenging task to organise a randomised, placebo-controlled study in headache types with low prevalence (51). Indomethacin-sensitive headache disorders can be difficult to treat when the subject does not tolerate them or has a contraindication for indomethacin (2).

#### Hemicrania continua

The first study reporting the efficacy of nVNS in other headaches described the effect on participants with hemicrania continua. In 2013, Nesbitt et al. published the test results of two patients who were previously responsive to stimulation of the occipital nerve. Both patients reported an improvement in the background pain and in painful autonomic exacerbations and would recommend the device to other patients (52). The subsequent case reporting a beneficial effect in indomethacin-responsive headaches was a 58-year-old patient with a long history of hemicrania continua. The patient noticed an immediate beneficial effect in daily headache severity, with a significant reduction from 8/10 to 5/10 in a numeric score, following the first stimulation. A reduction in painkiller consumption and an improvement in quality of life and anxiety and depression scores were also significant. The patient used a total daily dose of 540 s of stimulation, plus the possibility of 360 s more during acute attacks. This patient used the maximum intensity of stimulation permitted by the device (Table 2) (53). Unfortunately, this is the only study mentioning the intensity of VNS stimulation achieved during treatments.

**Table 2:** Participant's characteristics and stimulation parameters of studies assessing nVNS in rarer primary headaches.

Author (reference)	Year	Diagnosis (N)	Type of study	Female %	Age (SD)	Duration of stimulation	Stimulation parameters	Stimulation details
Nesbitt et al (52)	2013	HC (2)	Open Label	50%	56–61	24–32 weeks	NR	NR
Eren et al (53)	2016	HC (1)	Open Label	0%	58	4 weeks	Maximum 24 V and 60 mA DC sine wave series of 5kHz for 1 ms, repeated every 40 ms (25 Hz)	AA: patient reached maximum level 40/40
Tso et al (51)	2017	HC (9), PH (6)	Retrospective	73.30%	42.9	3 months to 5 years		NR
Trimboli et al (49)	2018	RTT CM (23), CCH (12), HC (4), SUNA (2)	Open Label	54%	44 (median)	> 3 months		AA. Training provided
Kamourieh et al (54)	2019	CPH (8)	Retrospective	37.50%	48	3–19 months		AA, MC, Training provided and technique reviewed
Moreno-Ajona et al (55)	2021	PCH (1)	Open Label	0%	53	>3 months		AA, MC, Training provided and technique reviewed

**Table:** 

Author (reference)	Acute Use	Preventive use	Pulses per dose	Seconds of stimulation	Stimulation intervals	Stimulation frequency	Total seconds	Location
Nesbitt et al (52)	Yes	NR	NR	NR	NR	NR	NR	NR
Eren et al (53)	Three pulses acutely	Morning and evening	3	90	NR	2	540/24 h + acute	Left
Tso et al (51)	NR	Yes	2–4	120	NR	2–3 (twice if 4 stimulations)	480–1,080/24 h	Ipsilateral to headache
Trimboli et al (49)	Three consecutive doses	Three times a day	2	90	Consecutive	3	540/24 + acute	Either one side or bilateral
Kamourieh et al (54)	No	Three times a day	2	120	Consecutive	3	720/24 h	Ipsilateral to headache
Moreno-Ajona et al (55)	No	Three times a day	2	120	Consecutive	3	720/24 h	Bilateral

Aa, amplitude adjustment; CCH, chronic cluster headache; CM, chronic migraine; CPH, chronic paroxysmal hemicrania; DC, direct current; HC, hemicrania continua; MC, muscle contraction; NA, not applicable; NR, not reported; PCH, primary cough headache; RTT, refractory to treatment

Tso et al. described the efficacy of VNS in a retrospective description of 14 patients, 8 with hemicrania continua and 6 with chronic paroxysmal hemicrania (51). Seven of the participants with hemicrania continua had a reduced severity of the continuous pain, and two of them reported reduced severity and duration of the exacerbations with acute treatment. One of the patients with paroxysmal hemicrania became free from attacks, and the other three patients reported a reduction in attack severity, two a reduction in frequency and one in duration. The patients received daily 480–1,080 seconds of treatment and were followed up between a period of 3 months and 5 years.

Four patients with hemicrania continua receiving acute pulses summated to 540 s of preventive stimulation per day were assessed by Trimboli et al. Continued treatment for 3 months was beneficial to two patients. One of them presented a 72.7% reduction in headache exacerbations and 1.8/10 in headache severity. However, exacerbations reduced of only 27.3% after 11 months. The second participant had a reduction from 10 to 1 day of exacerbations per month on the third month, which persisted at two monthly exacerbations after 10 months. Headache intensity reduced from 5.9/10 to 2.5/10 on the third month and persisted at 2.9/10 at the end of follow-up (49).

A more recent study by Kamourieh et al. found nVNS to be effective at 720 s per day, in reducing headache frequency in eight patients with chronic paroxysmal hemicrania, who had responded initially to indomethacin, but could not tolerate it, and had failed to respond to an average of three preventive medications. At 3 months of treatment, the mean monthly headache frequency had dropped 68%, and it had further dropped to 75% at the end of the follow-up (both *P *< 0.05). A 50% improvement in headache frequency was reached between 3 and 6 months of daily treatment. Headache severity also dropped 50%, 2/10 points in a verbal scale, after 3 months, and a further 2/10 points at the end of follow-up (8/10 to 4/10). Similarly, headache duration halved after 3 months. Anxiety and depression scores improved significantly (54).

#### Cough headache

A single patient with cough headache that was responsive to intramuscular indomethacin was reported to have headache freedom following preventive treatment with nVNS. After 1 month of treatment up to one stimulation three times a day, the patient started noticing an improvement in headache frequency, severity, and duration, from 10 attacks a day at 8/10 that lasted for 30 min to occasional attacks, rated 4/10 with a 10–20 min of duration. At two pulses three times a day, the patient became completely headache-free (55).

### Efficacy of nVNS in SUNA

Only the study of Trimboli et al. assessed nVNS in two patients with a diagnosis of SUNA, with the same stimulation parameters and doses described previously. These patients had between 10 and 600 attacks per day, rated 7–8/10 in severity. None of them reported any beneficial effect after 3 months on nVNS (49).

## Discussion

Most papers mentioned here have reported some kind of benefit in patients with long-lasting, debilitating primary headache disorders that were unresponsive to several lines of preventive treatments. For therapeutic studies, this corresponds with the level of evidence 4, which can be difficult to improve in disabling conditions with low prevalence. The reason why a cohort of patients responded better to stimulation than others with the same diagnosis is unknown. Device parameters, dose, or duration could play a key role in treatment efficacy, and given the clinical heterogeneity of these primary headaches, a careful tailoring of the treatment, personalised for every individual clinical presentation, could be considered.

In general, there has been incongruence regarding the different methodological designs of studies, as seen in the diverse range of electrical parameters reported in the pre-clinical models discussed herein. Likewise, human studies assessing VNS for heart failure presented a contrasting frequency and dose of stimulation (56). Similarly, these methods are heterogeneous in headache studies (Table 1). It has been proposed previously that individually adjusted doses could have influenced the treatment outcome (48).

One of the benefits of cervically applied nVNS is the possibility to regulate the intensity of the stimulation by the patient. Unfortunately, pain threshold has an inter-individual variability. Even though pain threshold can increase in a majority of patients following nVNS stimulation (57, 58), in more than one-third of them, it can diminish (58). This variability could be especially relevant in patients with a migrainous background (59), meaning that what a subject considers a tolerable intensity of stimulation could be unbearable for another. In view of the fact that a majority of people experience a headache throughout the course of their lives (60) and the high prevalence of migraine (61), a migrainous background, especially allodynic symptoms, should be considered when assessing tolerability to nVNS.

Among the proposed checklist of stimulation parameters to be reported in VNS, current (intensity) should be specified. When the option of selecting a dose is available, and there is titration to sensation or muscle response (such as lip pulling), the actual dose selected should be reported (62). Most studies described in this article included stimulation amplitude adjustments available in their methods, but the data regarding mA of output current have not been presented. Differences in efficacy among neuromodulation techniques in other primary headaches such as migraine (63) could be due to unstandardised settings and a lack of knowledge about the total doses of devices other than gammaCore. The therapeutic dose needed could also vary inter-individually and be age-dependent: younger patients may need higher output currents (64).

Another explanation for the disparity of results could be the treatment duration, which was also an important factor in epilepsy studies (65–67). Descending pain inhibition is not an immediate effect of nVNS (68). Studies on cluster headache assessed efficacy after 3 months of treatment (69). The results presented above represent an example that the latency of response to preventive treatment in primary headaches (70), also with neurostimulation, could be demonstrated after 3 months or above.

Also, patients with hemicrania who responded to treatment may need a significantly longer treatment schedule, as compared with those who did not respond in a retrospective cohort, and a higher number of total daily seconds (Villar-Martinez and Goadsby, unpublished results).

Adherence could also represent a main issue when assessing nVNS efficacy, as seen in migraine studies (71). Adherence should be considered in other primary headaches when assessing responsiveness.

A smaller and discrete device that uses transcutaneous auricular vagus nerve stimulation has been studied in migraine. The cutaneous distribution of the auricular branch of the vagal nerve is heterogeneous in cadaveric studies (72). However, the mechanism of action of auricular nerve stimulation also seems to target central pathways and structures involved in pain modulation. A significant modulation of activity in several brainstem areas was seen in functional MRI, including a decreased activity of the NTS, but also the noradrenergic locus coeruleus, serotonergic nuclei raphe, or parabrachial nuclei, and an increased connectivity with higher structures involved in the pain matrix, including cortical areas such as the temporoparietal junction or the somatosensory cortex (73). A randomised, double-blind monocentric clinical trial comparing low and high frequency of stimulation, showed promising results (74). At the time of the writing of this article, we could not find any literature referring to transcutaneous auricular vagus nerve stimulation for other primary headaches.

## Conclusion

Treatment with nVNS is well tolerated and efficacious in patients with other primary headache disorders, especially indomethacin-responsive headaches such as cough headache, hemicrania continua, and paroxysmal hemicrania. As with any preventive treatment, nVNS should be trialled for at least 3 months to consider any lack of efficacy, and assessment of adherence and correctness of the technique of stimulation should be granted before withdrawal. Training should be offered and the technique assessed in unresponsive patients. Tolerability could be inversely related to sensitisation in patients with a concomitant migrainous background. A more standardised way of reporting stimulation parameters is desirable for future studies.
